# Tracing transport of protein aggregates in microgravity versus unit gravity crystallization

**DOI:** 10.1038/s41526-022-00191-x

**Published:** 2022-02-17

**Authors:** Arayik Martirosyan, Sven Falke, Deborah McCombs, Martin Cox, Christopher D. Radka, Jan Knop, Christian Betzel, Lawrence J. DeLucas

**Affiliations:** 1 Institute of Biochemistry and Molecular Biology, Laboratory for Structural Biology of Infection and Inflammation, University of Hamburg, c/o DESY, Notkestrasse 85, Building 22A, 22607 Hamburg, Germany; 2 University of Alabama at Birmingham, Birmingham, AL USA; 3 The Aerospace Corporation, 5030 Bradford Drive, Bldg. 1, suite 220, Huntsville, AL 35805 USA; 4 St. Jude Children’s Research Hospital, Memphis, TN 38015 USA; 5 Institute of Plant Science and Microbiology, University of Hamburg, 22609 Hamburg, Germany

**Keywords:** Biochemistry, Biophysics

## Abstract

Microgravity conditions have been used to improve protein crystallization from the early 1980s using advanced crystallization apparatuses and methods. Early microgravity crystallization experiments confirmed that minimal convection and a sedimentation-free environment is beneficial for growth of crystals with higher internal order and in some cases, larger volume. It was however realized that crystal growth in microgravity requires additional time due to slower growth rates. The progress in space research via the International Space Station (ISS) provides a laboratory-like environment to perform convection-free crystallization experiments for an extended time. To obtain detailed insights in macromolecular transport phenomena under microgravity and the assumed reduction of unfavorable impurity incorporation in growing crystals, microgravity and unit gravity control experiments for three different proteins were designed. To determine the quantity of impurity incorporated into crystals, fluorescence-tagged aggregates of the proteins (acting as impurities) were prepared. The recorded fluorescence intensities of the respective crystals reveal reduction in the incorporation of aggregates under microgravity for different aggregate quantities. The experiments and data obtained, provide insights about macromolecular transport in relation to molecular weight of the target proteins, as well as information about associated diffusion behavior and crystal lattice formation. Results suggest one explanation why microgravity-grown protein crystals often exhibit higher quality. Furthermore, results from these experiments can be used to predict which proteins may benefit more from microgravity crystallization.

## Introduction

Proteins (we will use the term protein consistently to cover also other bio-macromolecules such as nucleic acids and protein-nucleic acid complexes as well) are vital and important macromolecules without which our bodies and other living organisms would be unable to repair, regulate, or protect against unwanted infectious organisms. Determination of the atomic three-dimensional structure of proteins provides critical information that allows scientists to understand how they function and interact. X-ray crystallography is the most efficient method to determine protein structures, although the technique requires growth of protein crystals of sufficient quality. Protein crystals grown in microgravity, initially utilizing unmanned rockets and subsequently on US space shuttle missions^1^, resulted in clear crystal quality improvements, reported for several investigations via X-ray diffraction analysis^2–10^. A microgravity environment results in protein crystals that are sometimes larger, provided sufficient growth time is available, but often it is the crystal’s quality that improves as evidenced by comparisons of diffraction resolution, mosaicity, signal-to-noise ratio of diffraction throughout the resolution range, temperature factors and final electron density maps^5,7,8,11^.

Past microgravity experiments have investigated two possible reasons for the improved crystal quality that is often observed. This investigation provides experimental data to address the two prevailing theories regarding why a microgravity environment sometimes yields protein crystals of superior quality. The following hypothesis was addressed:

Improved quality of microgravity-grown protein crystals is the result of two macromolecular characteristics that exist in a buoyancy-free, diffusion-dominated solution:

1. Slower crystal growth rates, due to slower protein transport to the growing crystal surface and formation of protein depletion zones around growing crystals.
2. Predilection of growing crystals to incorporate protein monomers versus larger protein oligomers, due to differences in transport rates.

Understanding transport processes of biomolecules is of substantial importance in the growth of crystalline materials from a heated melt or from aqueous solutions, as well as for recent investigations regarding transport phenomena of cellular phase separation and cellular organization of biomolecules^12–19^. Density differences develop near growing crystal surfaces, produced by the incorporation of molecules from solution into the crystalline lattice^20–26^. In a microgravity environment, associated with absence of convection, there is reduced mixing of the crystallization solution and the transport of protein molecules is dominated solely by diffusion. It has been suggested that while a crystal grows from solution, a reduction of the protein and salt molecules from the solution surrounding the crystal, generates a depletion zone resulting in regions of varying concentrations^27–29^. Zones with decreased protein concentration, lower than that of the bulk solution, are generated near the growing crystal and the crystal grows in an environment with a lower supersaturation. In a diffusion-controlled microgravity environment, the transport of protein molecules occurs in quasi-stable depletion zones and the protein molecules diffuse more slowly towards the growing crystal surface^30^. In addition, large protein aggregates diffuse more slowly than monomeric protein, thus the development of depletion zones may act as a diffusion filter that reduces the incorporation of aggregate impurities into the growing crystals.

**Fig. 1:**
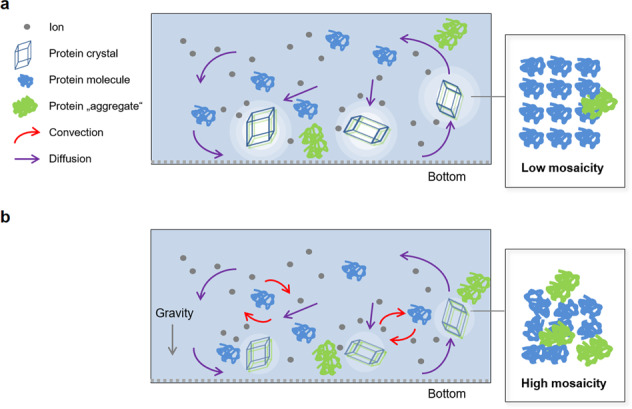
Scheme showing comparative mass transport. under microgravity and 1 G. **a** Under microgravity conditions, **b** on earth and resulting effects on protein crystal quality/mosaicity are shown. Particularly, the formation of spherical protein and protein aggregate depletion zones under microgravity conditions is supposed to be facilitated by the suppression of convection^48^.

In microgravity, buoyancy-minimization and reduction of convective flow conditions provide a quiescent environment, thereby slowing the process of protein crystal nucleation and subsequent growth^23,24,27^. It is believed that the diffusion-dominated environment causes crystals to grow more slowly since random diffusion of large protein molecules is the predominant factor responsible for their transport toward a growing crystal^31,32^. As a result, incoming protein molecules (approaching the crystalline lattice in random orientations) would have additional time to find the energetically favored and three-dimensional correct position at the surface of the growing crystal lattice. An electron microscopy study was performed on crystals grown at different rates for hen egg-white lysozyme with the resulting images showing an increase in crystalline step defects as growth rates increase^33,34^. In some areas along the crystal surface, veils or open spaces formed at increased growth rates—apparently due to defects preventing the next incoming protein molecule from forming lattice contacts in the defect region. As a result, adjacent layers of protein forming on the crystal surface eventually grew over the open region, leaving a void or veil within the crystals that is assumed to be filled with aqueous solvent^33,34^. Two theories based on protein transport rate differences in microgravity versus unit gravity have been proposed to explain the observed microgravity crystal improvements (Fig. 1). One theory is based on expected slower crystal growth rates due to slower protein transport to the growing crystal surface. The second theory proposes that there is an exclusion of larger protein aggregates incorporated into the growing crystal (aggregates produce crystal defects that can reduce crystal quality)^35–37^. The theoretical explanation is based on the expected faster transport of a single folded protein molecule as opposed to aggregates of two or more protein molecules. There are several previous ground-based studies that indicate that a diffusive environment improves crystal quality and reduces protein aggregate impurity incorporation into growing crystals. Vekilov et al. studied the effect of convective solute and impurity transport using forced solution convection for crystallization of lysozyme protein^35^. Dimers of covalently bound lysozyme were introduced into an otherwise monomeric lysozyme growth solution. Their results indicate that at lower flow rates, an enhanced supply of solute to the crystal interface results in an increase in step velocity and growth rate, whereas more rapid convective transport leads to a reduction in growth kinetics (step bunching). They also demonstrated that solution flow enhances the incorporation of impurities, i.e. lysozyme dimers, into growing lysozyme crystals. Several other investigations using various techniques suggest similar differences for impurity incorporation in a diffusive versus convective environment^28,38–40^. In one study, the effect of mass transport on the incorporation of crystal impurities and crystal quality, was evaluated using a ceiling crystallization method shown to virtually eliminate convective flow^29,41^. The results demonstrated that diffusive mass transport, versus convective transport, reduces impurity incorporation and enhances crystal quality. Thus, based on previous experiments, it is justified to consider that microgravity-grown crystals may benefit from slow protein transport, naturally selecting for protein monomers, or the main building blocks of the crystal lattice. This can be any distinct and stable protein oligomer, versus the unwanted protein aggregates (i.e. dimer, tetramer or larger non-specifically aggregated, or misfolded protein) that typically exist in a protein crystallization growth solution. However, one study did find that there were no impurity incorporation differences for lysozyme crystals grown in microgravity and in unit gravity in the presence of varying amounts of lysozyme dimer^42^.

In a diffusion-dominated environment, larger protein aggregates will travel significantly slower than smaller protein monomers thereby decreasing aggregate contamination in the crystal lattice. For an ideal case consisting of un-solvated spheres, D is proportional to one over M to the 1/3 and for a random coil macromolecule, D is proportional to one over M to the 0.5, where D is the translational diffusion coefficient and M is the molecular weight of the particle (Fick’s 1st law of diffusion corresponding to Eq. (1)^43^,

1 $$J_n = - D\frac{{dn}}{{dt}}$$

where J is the diffusion flux [mol m^−2^ s^−1^] depending on the particle gradient and further

2 $$D \sim M^{\frac{1}{3}}$$

The translational diffusion constant D [m^2^ s^−1^], corresponding to Eq. (2), depends on the Boltzmann constant k, temperature T [K], solvent viscosity η [Pa s] and molecular mass via the radius r [m] of the particles as following, according to the Stokes-Einstein Eq. (3):

3 $$D = \frac{{kT}}{{6\pi \eta r}}$$

Therefore, for globular proteins it can be approximated that the value of D is somewhere between these two values. If we choose a relatively small protein like lysozyme with an exponent estimated at 0.4, the monomer D is proportional to 1/14,000^0.4^ and the dimer D is proportional to 1/28,000^0.4^. Thus, the diffusion coefficient for the dimer results in an ~24% reduction. For a lysozyme tetramer the diffusion rate would decrease by ~43%. This suggests that the improvement in crystal quality may be partially due to the predilection for microgravity-grown crystals to contain a higher percentage of monomers versus a mixture of monomers and larger aggregates as larger aggregates will move progressively slower toward the growing crystal than their respective monomeric building blocks. It should also be mentioned that many proteins tend to be unstable over time, partly losing their three-dimensional structure via unfolding during a crystallization experiment. The C- and N- terminal regions of a protein peptide chain are particularly prone to such effects occurring over time. A partially unfolded, in the worst case completely unfolded protein occupies substantially larger volumes than folded protein. For example, random coil peptide aggregates will also move substantially slower in solution under microgravity conditions and therefore, will be incorporated in growing crystals less frequently than their folded counterparts. In this context it can be concluded that crystallization experiments under microgravity may show improvement.

**Fig. 2:**
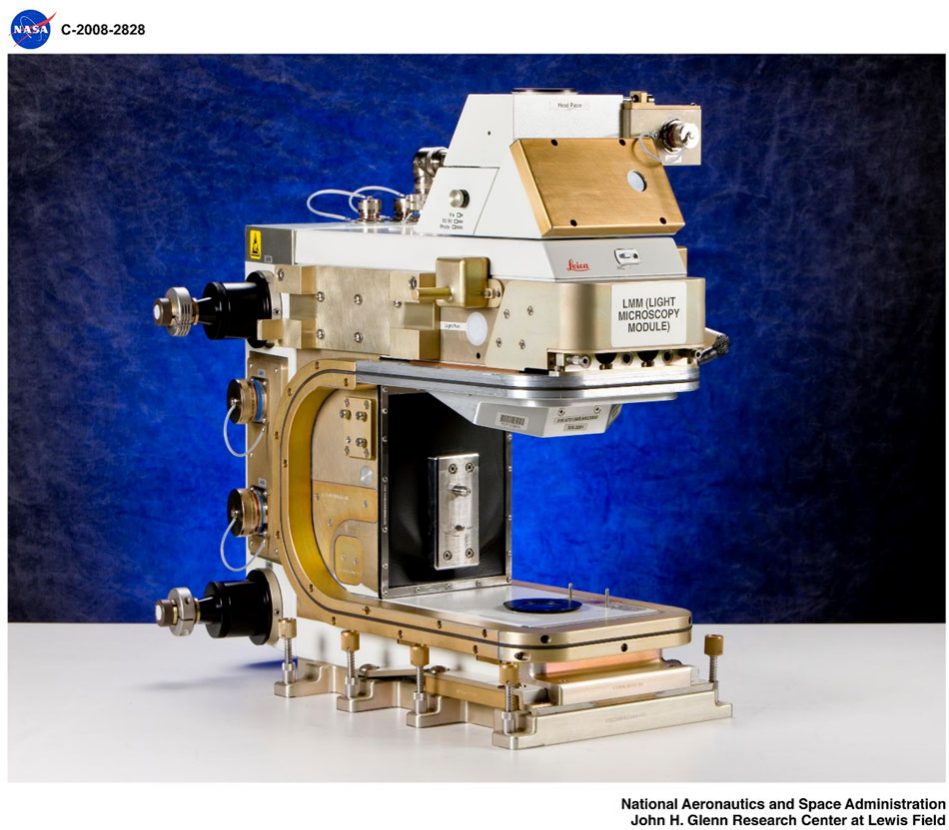
Light Microscopy Module (LMM). The LMM was used to visualize protein crystals on the International Space Station (ISS). The microscope was used to photograph the crystals at low (×5) and higher (×10) magnifications using white light and fluorescence microscopy. This image was kindly provided by NASA Glenn Research Center (GRC) and approved for use in this publication by NASA GRC.

**Fig. 3:**
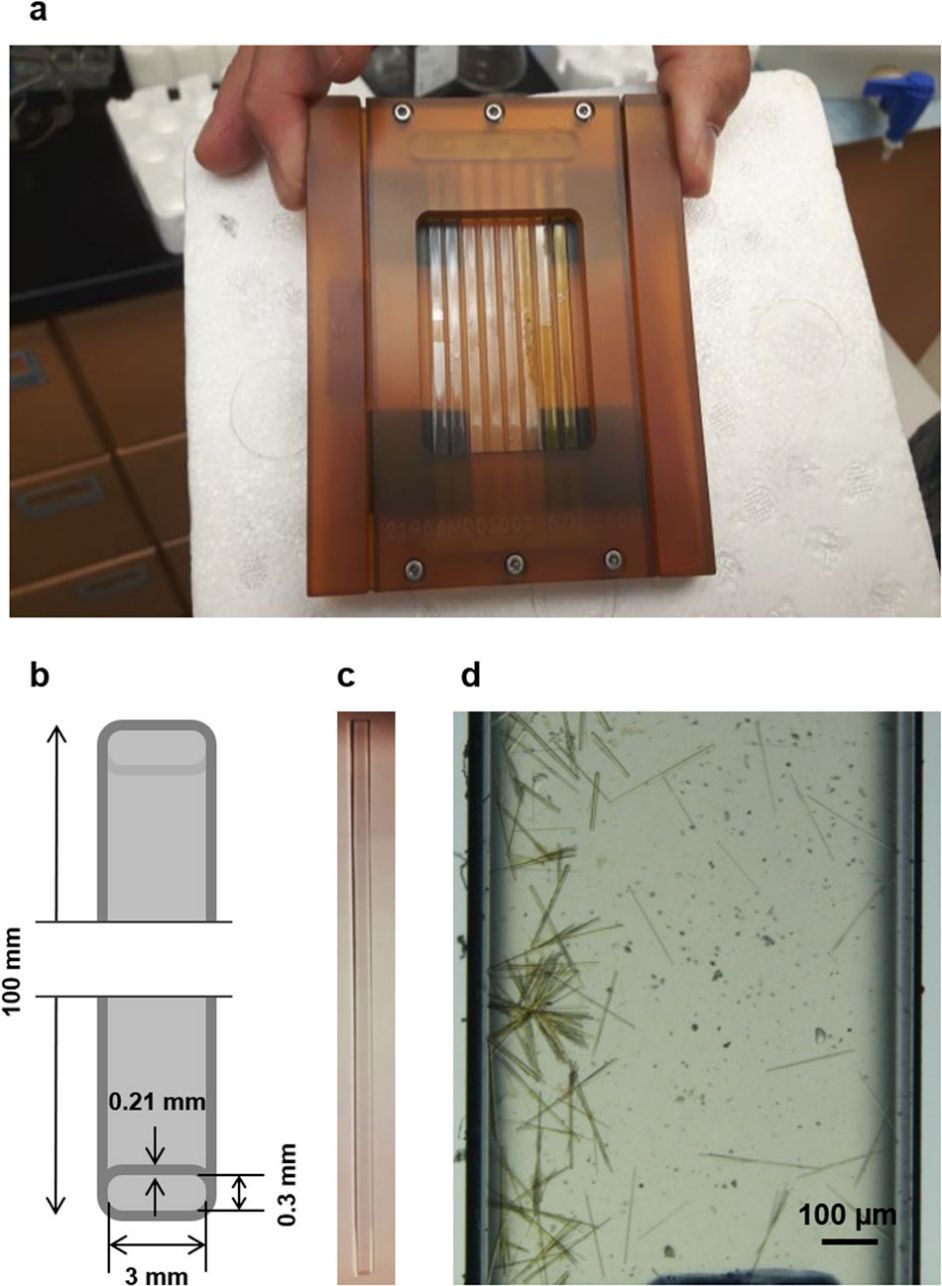
Crystallization experiment hardware. **a** The SPX11 flight cassette contained eight capillaries: capillaries 1–3 contain lysozyme, capillaries 4 and 5 contain bovine serum albumin and capillaries 6–8 contain *Pf*GST. **b** Schematic diagram illustrating the capillary shape with dimensions indicated. **c** Empty capillary. **d** Section of a capillary with grown *Pf*GST crystals.

We utilized the International Space Station’s Light Microscopy Module (LMM), shown in Fig. 2, and fluorescence labeling to address the two hypotheses mentioned before and previous experimental data regarding microgravity’s effect on protein crystal growth. Specially designed cassettes (Fig. 3), each containing eight capillaries, were used for the crystal growth and imaging experiments. Three proteins, chicken egg-white lysozyme, bovine serum albumin (BSA) and *Plasmodium falciparum* glutathione-*S*-transferase (*Pf*GST), were selected for the experiments.

## Results

### Crystal growth and morphology

**Fig. 4:**
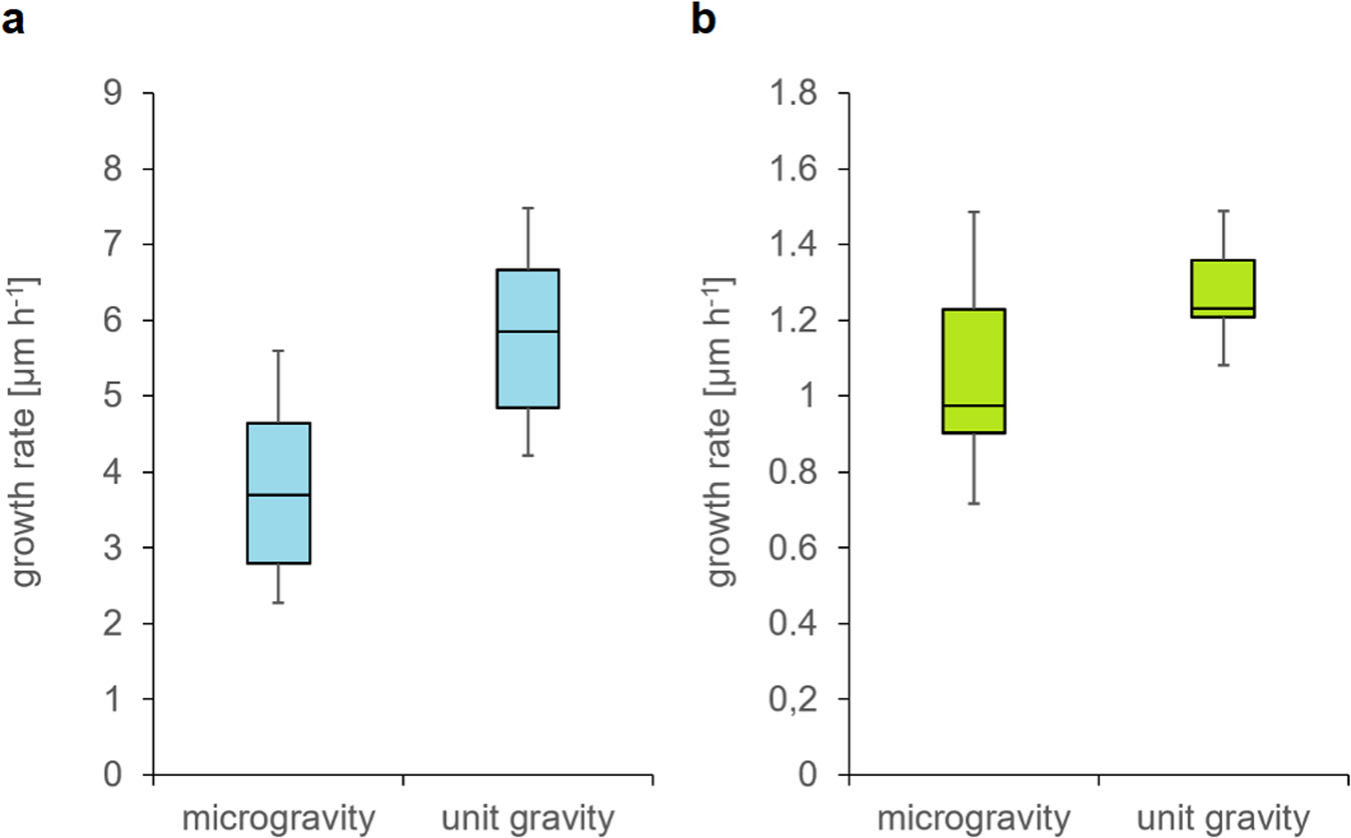
Comparison of the growth rates. **a** Growth rates are shown for lysozyme and **b** for *Pf*GST crystals at unit gravity and under microgravity conditions for the time frame from 5 to 147 h after thawing. For both conditions 17 crystals from the same corresponding capillary section were investigated.

**Fig. 5:**
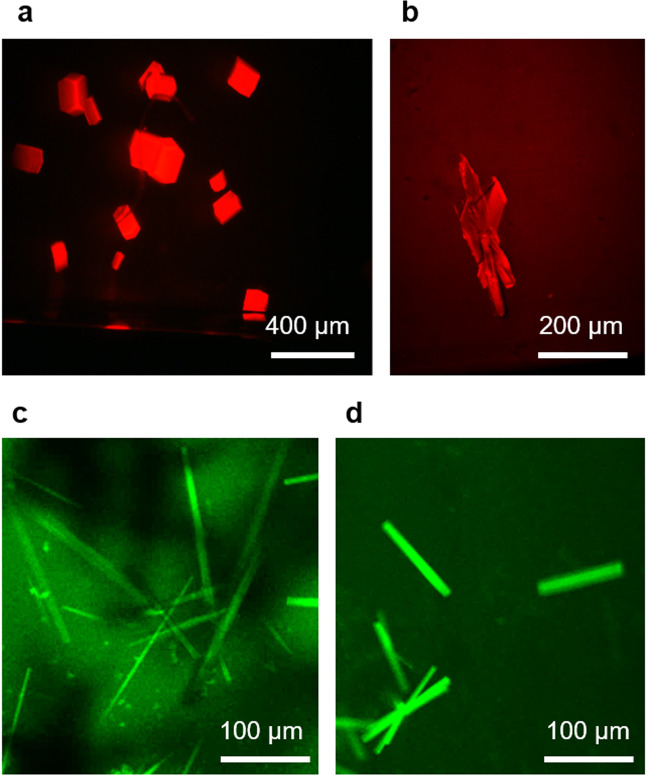
Representative confocal fluorescence micrographs. **a** Lysozyme and **b** BSA crystals in the presence of 5% dimer of the respective protein labeled with Alexa Fluor 594 NHS ester grown at 1 G. Representative *Pf*GST crystals were grown in the presence of **c** 1% tetramer impurity and **d** 5% tetramer impurity labeled with Alexa Fluor 488 TFP ester and the displayed crystals were also prepared under unit gravity conditions. The crystals from the capillaries utilized for further investigation were similar in size, which means ~180–220 µm in major axis length for *Pf*GST, 80–120 × 80–120 × 250–300 µm for BSA and 120–180 µm in all three directions for lysozyme. Fluorescence analysis was performed on equal crystal volumes thereby eliminating the influence of crystal size on total fluorescence.

A detailed statistical comparison of growth rates and crystal sizes along the different growth axes and in different sectors of the capillaries for microgravity and unit gravity crystallization was presented previously^44,45^ and therefore is not the focus of this publication. In summary, it was found that the average growth rate of the major axis of needle-shaped *Pf*GST crystals and lysozyme crystals was found to be higher (*p* < 0.01) at unit gravity compared to the microgravity environment. Figure 4 shows this comparison. Results from comparative crystal growth investigations showed a clear decrease in crystal growth rates along the major axis for microgravity-grown crystals of lysozyme and an increase in the length of the major axis for *Pf*GST crystals. The increase in length of the major axis for the *Pf*GST crystals is expected in a diffusion-dominated environment due to their needle-like shape. The leading edge of needle-like growing crystals receives new protein molecules via random diffusion from the front and sides, whereas the sides of needle-like crystals only receive new protein molecules from one side. In addition, the approximate number of crystals was not systematically changed under either condition, plus there was no indication for another crystal shape differing from the representative micrographs in Fig. 5 for either of the three proteins.

### Fluorescence microscopy and spectroscopy

A detailed comparison of impurity (aggregate) incorporation into growing chicken egg-white lysozyme, BSA and *Pf*GST crystals is presented. To investigate impurity incorporation into growing crystals for each protein, fluorescently labeled protein aggregate samples were prepared containing different protein aggregate ratios. Figure 5 shows typical fluorescence images obtained using a ground-based Zeiss LSM710 confocal laser scanning microscope.

**Fig. 6:**
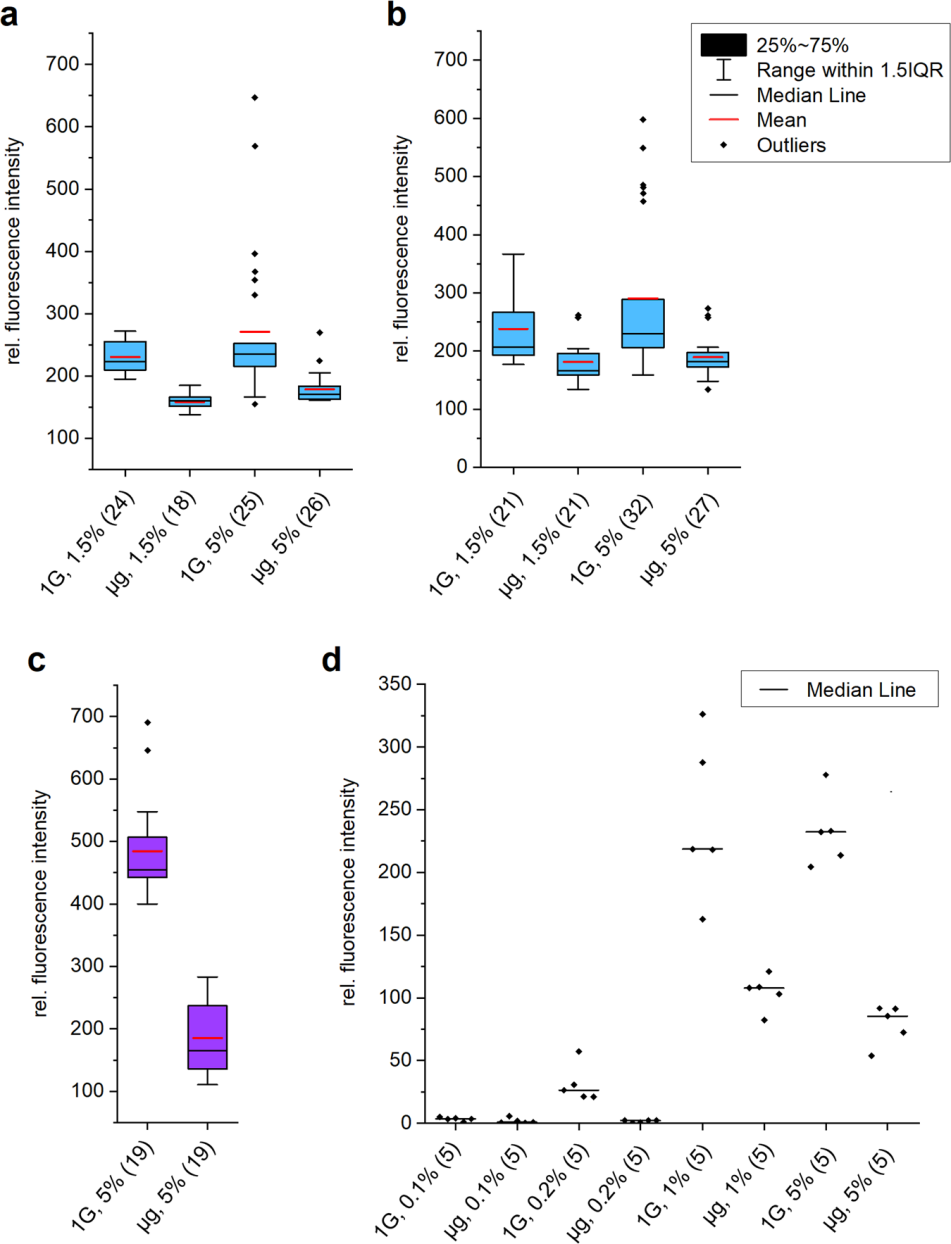
Spectroscopic quantification of fluorescent oligomers incorporated into crystals. Crystal aggregate incorporation in microgravity versus unit gravity control experiments is displayed. The box plots (**a**, **b**, and **c**; with symbols explained for each boxplot in **b**) show the aggregate incorporation based on fluorescence intensity analysis, for **a** lysozyme (SPX10 mission), **b** lysozyme (SPX 15 mission) and **c** BSA (SPX15 mission). For **d** *Pf*GST (SPX10 mission) all individual data points are included for clarity due to the lower number of crystals under investigation for each experimental condition. Individual data points are fluorescence intensities of crystals either grown at unit gravity (1 G) or microgravity (µg). The number of crystals analyzed under the respective conditions is provided in brackets and the relative amount of aggregates in % is provided as well.

As noted in the Materials and Methods section, the mean intensity distribution of a defined crystal volume can be measured, recorded and normalized. This allows comparison of intensities of an equal crystal volume, thereby enabling accurate comparison of microgravity versus unit gravity ground-control crystals. Quantitative analysis via the fluorescent microscopy software clearly confirmed a difference in aggregate incorporation for unit gravity versus a microgravity environment. Comparison of the mean fluorescence intensity of crystals normalized to the crystal volume showed differences between the microgravity versus unit gravity control crystals for all three proteins (albeit the difference for the smallest protein, lysozyme was rather small, with exception of some outlier data). As shown in Fig. 6, for lysozyme, BSA and glutathione-*S*-transferase, the recorded fluorescence intensities for the unit gravity control crystals are higher than for the corresponding microgravity-grown crystals using identical protein batches, prepared at the same time as the flight samples.

This suggests that our second hypothesis, predilection of growing crystals to incorporate protein monomers versus higher protein aggregates due to differences in transport rates, may play a role in the improved quality and size of microgravity-grown crystals. The SPX10 lysozyme fluorescence data (Fig. 6a) shows the average measurement for 24 ground-control crystals compared with 18 flight crystals with 1.5% fluorescent aggregate added in the growth solutions and 25 ground-control crystals compared with 26 flight crystals with 5.0% fluorescent aggregate added in the growth solutions. The fluorescence intensities determined for lysozyme crystals of the SPX15 mission (Fig. 6b) represent the average values for 21 ground-control crystals compared with 21 flight crystals with 1.5% fluorescent aggregate added in the growth solutions and 32 ground-control crystals compared with 27 flight crystals with 5.0% fluorescent aggregate added to the growth solutions. Similar to lysozyme crystal samples of SPX15, the crystals samples of SPX10 are equally reduced in fluorescence when grown under microgravity.

Moreover, the SPX15 BSA fluorescence data (Fig. 6c) shows the average measurement for 19 unit gravity control crystals compared with 19 microgravity-grown crystals with 5% fluorescent aggregate added in the growth solutions. The SPX10 glutathione-*S*-transferase fluorescence data (Fig. 6d) shows comparison of the incorporation of aggregates in 5 unit gravity controls versus 5 microgravity-grown crystals for each aggregate concentration, using identical protein batches.

Overall, quantitative analysis of equal volumes of crystals of approximately similar size via the fluorescence microscopy software demonstrates a difference in aggregate incorporation in a unit gravity versus microgravity environment for the three different protein crystallization experiments studied. Microgravity and unit gravity experiments were performed in parallel and activated at the identical time. Using a student *t*-test, it is indicated that for the aggregate concentrations 0.2%, 1 and 5% (*p* < 0.01), *Pf*GST crystals grown under microgravity possess a significantly lower fluorescence and thereby lower aggregate incorporation, within a confidence interval of 99%. Although, there is no significant difference in this comparison for the lowest aggregate concentration of 0.1% (*p* > 0.05). Further, there is a significant difference for both BSA and lysozyme, including both aggregate concentrations, with a confidence *p* < 0.01, when comparing the data plotted in Fig. 6 in a *t*-test. However, as the data is not normally distributed, we further selected an appropriate nonparametric Mann–Whitney-*U* test^46^ here, which indeed confirmed the statistical significance for both BSA and lysozyme with a significance level of 0.05, contributing to the hypothesis of reduced aggregate incorporation under microgravity. Using a Mann–Whitney-*U* test^46^, it was further verified that for the aggregate concentrations 0.1%, 0.2%, 1 and 5% (*p* < 0.05), *Pf*GST crystals grown under microgravity possess a significantly lower fluorescence and thereby lower aggregate incorporation.

### X-ray diffraction

*Pf*GST, a highly attractive target for the development of anti-malaria compounds in medicine, was crystallized, for the first time, under microgravity conditions. Unit gravity and microgravity-grown crystals were used for comparative X-ray diffraction data collection. All *Pf*GST crystals analyzed, not only share the same morphology but also belong to the same space group, P2_1_. A substantial improvement in average mosaicity and resolution was observed for the microgravity-grown crystals. On average, there is an improvement of the maximum resolution, R-factors, I/σ(I) value, as well as a clear reduction of mosaicity for microgravity-grown crystals in the absence of tetramer impurity, based on averaging three crystal datasets (Table 1). Independent from the maximum resolution, the average I/σ(I) for an identical resolution range is increased under microgravity conditions compared to the unit gravity samples (Tables 1 and 2). The highest maximum resolution observed among the datasets was ~2 Å for a crystal grown under microgravity. Furthermore, at two of three different selected concentrations of tetramer impurity (0.2 and 5%), the average value of crystal mosaicity is lower and the average number of obtained unique reflections is higher for the corresponding crystals grown under microgravity conditions. The average maximum resolutions of the respective datasets are improved for the microgravity-grown crystals. Nonetheless, in agreement with fluorescence quantification data, the average mosaicity is increasing for crystal growth conditions with higher amounts of fluorescently labeled tetramer added to the crystallization solution, corresponding to higher amounts of incorporated tetramer. X-ray diffraction statistics are summarized in more detail in Tables 1 and 2.

**Table 1:** Comparative summary and statistics of *Pf*GST X-ray diffraction data with 0 and 0.2% fluorescence labeled tetramer applied as impurity during the crystallization experiments respectively.

*Pf*GST				
Environment	Ground	Microgravity	Ground	Microgravity
Relative amount of tetramer [%]	0	0	0.2	0.2
Number of datasets	3	3	3	3
Temperature [K]	100	100	100	100
Wavelength [nm]	0.9919/0.9919/0.9919	0.9919/0.9919/0.9919	1.0332/1.0332/1.0332	1.0332
Space group	P2_1_	P2_1_	P2_1_	P2_1_
Unit cell parameters				
a [Å]	61.3/61.2/61.5	61.6/61.5/61.4	61.2/61.2/61.3	60.1/60.1/61.1
b [Å]	69.8/69.6/69.8	69.7/69.9/69.8	69.5/69.8/69.6	69.3/69.3/69.6
c [Å]	98.8/100.3/100.7	101.8/100.6/99.6	99.5/100.2/99.9	98.5/99.4/98.8
β [°]	90.2/90.1/90.2	92.4/92.4/91.9	92.2/92.3/92.2	91.9/92.3/91.8
Total number of reflections	38,264 49,046 72,528	16,4488 45,729 125,680	142,274 101,249 106,086	113,202 138,518 145,519
Unique number of reflections	11,385 14,755 14,429	48,857 13,653/ 37,211	24,899 14,813 15,441	16,505 20,080 21,494
Resolution range [Å]	50.00–3.42 (3.62–3.42) 50.00–3.14 (3.33–3.14) 50.00–3.19 (3.38–3.19)	50.00–2.12 (2.25–2.12) 50.00–3.24 (3.43–3.24) 50.00–2.30 (2.44–2.30)	50.00–2.63 (2.79–2.63) 50.00–3.14 (3.33–3.14) 50.00–3.10 (3.28–3.10)	50.00–3.00 (3.18–3.00) 50.00–2.82 (2.99–2.82) 50.00–2.76 (2.92–2.76)
R_meas_ [%]	7.9 (55.9) 16.2 (58.2) 14.9 (55.3)	9.9 (56.6) 18.6 (56.8) 8.0 (60.1)	11.5 (55.5) 18.0 (52.7) 20.4 (53.7)	12.8 (52.4) 13.2 (53.69) 12.0 (53.6)
R_symm_ [%]	6.6 (47.4) 13.6 (48.4) 13.4 (49.6)	8.3 (49.9) 15.6 (49.5) 6.7 (50.3)	10.4 (50.2) 16.6 (48.7) 18.9 (49.7)	11.9 (48.5) 12.2 (49.7) 11.1 (49.4)
Mean I/σ(I)	12.44 (2.49) 7.57 (2.28) 9.96 (3.28)	12.09 (2.57) 7.47 (2.45) 12.82 (2.30)	13.97 (3.22) 10.03 (3.65) 8.78 (3.33)	13.81 (4.12) 13.70 (3.83) 12.05 (3.91)
Mean I/σ(I) (res. range 50.00–3.45 Å)	13.45 ± 3.74	20.48 ± 6.07	11.15 ± 4.55	17.12 ± 2.06
CC_1/2_	99.9 (94.4) 99.7 (86.8) 99.7 (88.1)	99.8 (83.4) 99.6 (94.9) 99.8 (85.6)	99.8 (91.6) 99.6 (92.9) 99.2 (91.9)	99.8 (94.5) 99.8 (93.8) 99.8 (94.4)
Average mosaicity [°]	0.14/0.34/0.27	0.12/0.11/0.12	0.12/0.12/0.07	0.34/0.15/0.23
Completeness [%]	99.2 (98.9) 98.8 (96.3) 99.7 (99.7)	99.5 (99.4) 98.9 (96.7) 99.4 (98.8)	99.2 (98.7) 99.3 (97.1) 99.7 (99.6)	98.6 (95.0) 99.4 (98.9) 99.6 (97.9)

For each condition values for three diffraction datasets are provided and values in parenthesis refer to the outer resolution shell.

**Table 2:** Comparative summary and statistics of *Pf*GST X-ray diffraction data using 1 and 5% fluorescence labeled tetramer applied as impurity during the crystallization experiments respectively.

*Pf*GST				
Environment	Ground	Microgravity	Ground	Microgravity
Relative amount of tetramer [%]	1	1	5	5
Number of datasets	3	3	3	3
Temperature [K]	100	100	100	100
Wavelength [nm]	1.0089/1.0089/1.0089	1.0089/1.0089/1.0089	1.0089/1.0089/1.0089	1.0089/1.0089/1.0089
Space group	P2_1_	P2_1_	P2_1_	P2_1_
Unit cell parameters				
a [Å]	61.4/61.1/61.8	60.7/60.1/61.4	61.3/61.4/61.4	61.8/60.4/61.3
b [Å]	69.7/69.6/69.9	69.3/69.9/69.9	70.2/70.0/70.5	70.2/69.4/69.7
c [Å]	100.6/98.7/102.5	97.6/99.5/99.4	101.2/97.6/99.0	99.4/94.2/99.6
β [°]	91.1/92.2/92.6	89.9/91.2/91.7	92.2/91.9/91.6	91.7/89.8/92.2
Total number of reflections	32,777 27,846 71,134	37,077 29,252 10,4867	31,396 48,626 41,703	68,641 39,688 155,774
Unique number of reflections	9870 9467 40,196	21,271 9039 15,536	10,453 27,761 24,230	39,050 22,787 32,601
Resolution range [Å]	50.00–3.53 (3.75–3.53) 50.00–3.59 (3.81–3.59) 50.00–2.73 (2.90–2.73)	50.00–3.27 (3.47–3.27) 50.00–3.62 (3.84–3.62) 50.00–3.09 (3.28–3.09)	50.00–3.48 (3.69–3.48) 50.00–3.08 (3.26–3.08) 50.00–3.23 (3.42–3.23)	50.00–2.74 (2.91–2.74) 50.00–3.17 (3.37–3.10) 50.00–2.41 (2.55–2.41)
R_meas_ [%]	29.4 (57.9) 29.4 (63.8) 17.2 (68.9)	23.2 (64.9) 23.3 (58.1) 10.1 (54.9)	19.6 (54.9) 10.9 (68.5) 14.9 (69.6)	20.5 (64.6) 15.5 (66.4) 7.5 (53.9)
R_symm_ [%]	24.6 (48.0) 24.0 (52.1) 12.2 (48.8)	16.5 (46.0) 19.4 (48.1) 9.3 (50.7)	16.0 (44.9) 7.8 (49.2) 10.7 (50.4)	14.7 (46.3) 11.1 (47.8) 6.7 (48.0)
Mean I/σ(I)	4.07 (2.33) 4.35 (1.71) 6.75 (1.59)	5.38 (1.46) 4.82 (1.90) 14.88 (3.82)	5.91 (2.36) 7.93 (2.01) 5.35 (1.43)	3.91 (1.77) 5.09 (1.37) 14.42 (3.05)
Mean I/σ(I) (res. range 50.00–3.45 Å)	5.15 ± 2.10	6.19 ± 4.23	5.94 ± 2.11	11.21 ± 9.40
CC_1/2_	97.2 (69.0) 97.4 (77.7) 98.3 (75.7)	97.4 (79.6) 99.3 (92.2) 99.9 (96.3)	91.1 (98.6) 99.6 (71.6) 99.0 (77.3)	98.1 (82.0) 98.8 (64.5) 99.9 (92.0)
Average mosaicity [°]	0.37/0.44/0.51	0.77/0.61/0.25	0.48/0.52/0.73	0.25/0.65/0.15
Completeness [%]	94.1 (74.4) 96.2 (89.7) 88.4 (85.7)	87.1 (70.3) 92.9 (60.2) 99.4 (97.0)	93.5 (74.2) 92.0 (79.6) 90.7 (79.8)	89.5 (80.4) 88.3 (61.5) 99.5 (98.2)

Values for three diffraction datasets are provided and values in parenthesis refer to the outer resolution shell.

## Discussion

Our investigations addressed two prevailing theories regarding why microgravity-grown protein crystals often exhibit improved X-ray diffraction statistics compared to unit gravity control crystals. The first theory concerning differences in crystal growth rates found in our experiments was previously addressed^44,45^. The reduction in growth rate under microgravity versus unit gravity can be explained by the driving force ratio of diffusion as defined by Tanaka et al.^47,48^. Data from SPX10 as well as previous studies demonstrated differences in the growth rate and geometry of crystals grown in microgravity versus unit gravity control experiments^44,45,49^. However, for the crystals under investigation, as mentioned previously, changes were not observed for shape and geometry for microgravity versus unit gravity crystals.

Results presented here address the second theory: “Predilection of growing crystals to incorporate protein monomers versus higher protein aggregates due to differences in transport rates”. Previous studies using cryo-electron microscopy and atomic force microscopy revealed severe defects and dislocations in protein crystals^33,34^. Several studies demonstrate how small molecule and macromolecular impurities cause defects and dislocations in protein crystals^50–54^. Protein crystals affected by such defects are often completely disordered, thereby not fulfilling Braggs law^55^ and not allowing generation of diffraction intensities, other than background scatter. Defects and dislocations reduce the number of productive scattering units which is proportional to the overall diffraction intensity, while in parallel increasing diffuse scattering or background noise. In addition, defects may also affect the ultimate crystal size, its dimensions and growth cessation, which may explain the observation that crystals grown in microgravity are in some cases larger than their unit gravity counterparts^3^.

These investigations used monomeric and aggregate populations of three proteins, chicken egg-white lysozyme, BSA and *Pf*GST. Fluorescent dyes (Alexa fluor 594 for lysozyme and BSA and Alexa fluor 488 for glutathione-*S*-transferase) were covalently attached to stable aggregate populations for each protein. Crystallization experiments were prepared such that protein samples used for the comparative crystallization experiments contained different protein aggregate ratios. This allowed examination of the percent incorporation of aggregate into growing protein crystals since the monomeric form of each protein did not contain fluorescent dye. As can be seen from Fig. 6, fluorescent analysis of equal crystal volumes for each protein showed significantly more aggregate in the control crystals (grown at unit gravity) compared to the microgravity-grown crystals. On flight SPX10, for lysozyme, the amount of average aggregate incorporation (1.5% aggregate supplementation) was ~40% higher and the average aggregate incorporation (5% aggregate supplementation) was ~60% higher for the unit gravity-grown crystals. On flight SPX15, for lysozyme, the amount of aggregate incorporation (1.5% aggregate supplementation) was ~38% higher and the aggregate incorporation (5% aggregate supplementation) was also ~60% higher for the unit gravity-grown crystals. However, there were several data points at significantly higher fluorescent intensities that may have incorrectly prejudiced these results. The box plot in Fig. 6 shows only small differences between the microgravity and unit gravity crystal fluorescence when these outliers are not considered, despite the student *t*-test and Mann–Whitney-*U* test^46^ results. For BSA, the amount of aggregate incorporation (5.0% aggregate supplement in the solution) was ~170% higher for the unit gravity-grown crystals.

*Pf*GST crystals grown at unit gravity contain a higher amount of tetramer impurity compared to the crystals grown under microgravity for all the tetramer concentrations studied (Fig. 6). For all tetramer concentrations higher than 0.1%, the average fluorescence of the unit gravity-grown crystals increased by at least 100%. Up to a concentration of 1% tetramer the total fluorescence of the unit gravity-grown crystals is continuously increasing. Similarly, the fluorescence of the microgravity-grown crystals reaches its maximum at 5%. This trend was also observed for the mosaicity and X-ray diffraction experiments when comparing the setups with 1 and 5% tetramer. The mosaicity increases with increasing tetramer aggregate concentration from 0.2 to 5% under terrestrial conditions, indicating additional incorporation of tetramer. The mosaicity is nearly 50% reduced under microgravity for 5% tetramer content but increased under microgravity conditions for 1% tetramer content compared to the unit gravity environment. Reduced mosaicity of macromolecular crystals under microgravity in general is, as discussed, in agreement with several previous experiments^3,56^, although these previous experiments did not apply a defined gradual addition of impurities with fluorescence-based monitoring.

Notably, according to Tanaka et al.^47,48^, a theoretical discussion of the reduction in impurity incorporation was presented as dependent on the ratio of the kinetic parameter β of the impurity (β_i_) and the target protein in the crystal lattice as well as on the ratio of the diffusion constant, D, of the impurity (D_i_) and the target protein. Therefore, considering almost identical affinity of aggregate and monomer protein for the growing crystal and structural similarity of monomer protein and supplemented aggregate, a reduction of aggregate incorporation into the crystal would be expected since the aggregates have a lower diffusion constant. On the other hand, smaller impurities, which could be fragments of the target protein, could be depleted accordingly, provided their affinity is lower. Considering a relatively large protein crystal, probably close to its maximal size, as the depletion zone effectiveness is increasing with increasing crystal size, the impurity uptake filtration was described to tend to approximate $\frac{{\beta \;D_i}}{{\beta _i\;D}}$. Consequently, an accurate experimental determination of the values for β and D for the three different proteins under investigation, might, according to the proposed model, reflect the different relative fluorescence reduction of the microgravity-grown crystals. As observed in Fig. 6, the reduction of the mean and median value of fluorescence, when comparing microgravity-grown with unit gravity-grown crystals, is relatively low for lysozyme in comparison to BSA and *Pf*GST. However, additional parameters including the crystal size would need to be considered for a more valuable comparison of the different proteins.

Further quantification and verification of depletion zones of the growing crystal depending on the local diffusion coefficient, crystal size as well as affinity of monomeric protein and impurity to the crystal lattice of a selected protein, as modeled by Tanaka^48^, would be informative. Complementary experiments could be performed in batch due to the comparatively simplified crystal growth kinetics^48,57,58^.

In summary, the results suggest that microgravity supports growth of higher quality protein crystals, via slower mass dependent protein transport in a diffusion-dominated environment. Slower growth rates could be a consequence of slower protein transport or lower supersaturation close to the crystal surface. The results support both theories presented in the hypothesis: (1) crystal growth rates are generally slower thereby allowing approaching protein molecules time to become uniformly incorporated in the crystalline lattice^44,45^ and (2) the diffusion-dominated environment acts as a filter in that monomeric forms of the protein will be transported to the growing crystalline lattice faster than aggregated protein, which possesses a smaller diffusion constant due to the higher hydrodynamic radius. In this context it can be concluded that higher molecular weight proteins should demonstrate a certain degree of increased benefit due to larger differences in transport rates for monomers versus aggregates, i.e. large impurities. These results suggest that information about the presence of unwanted aggregates, or fractions of partially unfolded proteins in purified protein preparations could be used in the selection process for proteins likely to exhibit additional benefit from microgravity crystallization. Purified protein samples even with minimum aggregation propensity and typically minor amounts of trace impurities exhibit additional benefit from microgravity crystallization. In conclusion, the data from Spx10 and Sps15 for all three proteins appears to confirm the second hypothesis (predilection of growing crystals to incorporate protein monomers versus larger protein oligomers in a microgravity environment) thereby agreeing with several previous investigations.

## Methods

### Sample preparation

Three different proteins, monomeric hen egg-white lysozyme, M.W. ~14.4 kDa, monomeric BSA, M.W. ~66.5 kDa, and dimeric *Pf*GST, M.W. ~56 kDa, were used for the experimental investigations. *Pf*GST from *Plasmodium falciparum* was expressed and purified in preparation for crystallization as previously reported with few modifications. Recombinant gene overexpression was performed in *E. coli* BL21(DE3) cells in overnight cultures at 19 °C. Cells were harvested by centrifugation and resuspended in Dulbecco’s phosphate-buffered saline (pH 7.4). The cell suspension was sonicated, and cell debris was removed by centrifugation at 17,000 × *g* at 4 °C for 1 h. Recombinant *Pf*GST was purified applying gravity-flow glutathione affinity chromatography resin, additionally, *Pf*GST dimers and tetramers were separated by size-exclusion chromatography by utilizing a HiLoad 26/600 Superdex 200 column (GE Healthcare) for subsequent labeling of the tetrameric protein (V_elution_ ≈ 170 mL). More details about the dimeric and tetrameric state of the protein and its transition were provided by Perbandt et al.^59^. To obtain pure and stable dimeric *Pf*GST for crystallization trials and to stabilize the dimeric state, the protein was eluted from the affinity chromatography column using 10 mM of the GSH analog *S*-(*p*-bromobenzyl)-GSH, which was synthesized and applied according to Vince et al.^60^. 4-Bromobenzyl bromide was dissolved in 500 mL of 50% ethanol aqueous solution to a concentration of 12 mM in a single neck round-bottom flask. Then L-glutathione was added into the solution via vigorous stirring. After 4-bromobenzyl bromide and L-glutathione were completely dissolved, 5 M sodium hydroxide was added dropwise into the mixture and pH 9.2 was maintained under stirring at RT for 3 h. Then, 10 M hydrochloric acid was slowly added into the mixture until reaching pH 2.1. The obtained solution was removed into a 1 L round flask and the excess of solvent was removed in a rotary evaporator at RT and 175 mbar to get a white solid product. The white solid product was dissolved in sodium phosphate buffer. Then, 1 M NaOH was added dropwise to the solution to reach pH 6.7. The final solution with pH 6.7 was sterile filtered.

The dispersity and particle radius distribution of *Pf*GST dimers (30 mg/mL), as utilized for crystallization trials, were analyzed at 20 °C applying dynamic laser light scattering (DLS) to verify and confirm the stability of the oligomeric state over time for five consecutive days utilizing a DLS SpectroLight 300 system (Xtal Concepts GmbH, Hamburg, Germany). Time-resolved DLS measurements verified the mono-dispersity of the *Pf*GST dimer with R_H_ = 3.7 ± 0.1 nm and tetramer, which was subsequently fluorescence tagged and used as defined aggregate, with R_H_ = 5.7 ± 0.1 nm, respectively, averaged over time.

Monomeric chicken egg-white lysozyme was obtained by subjecting commercially obtained protein (Biomedical’s LLC) to size-exclusion chromatography (Yarra-3000 size exclusion column). To produce stable aggregates, the purified monomeric protein was further subjected to cross-linking by using glutaraldehyde as a cross-linking agent. One gram of the purified protein was crosslinked in 100 mL 0.5% (w/w) glutaraldehyde in phosphate buffer (0.055 M Na_2_HPO_4_, 0.09 M NaH_2_PO_4_, pH 7.0) for 2 h at 22 °C. The cross-linked protein was then reapplied to the Yarra-3000 size exclusion column equilibrated with 50 mM acetate buffer pH 4.8, to separate dimeric lysozyme from remaining monomeric protein. The monomeric protein and dimer were concentrated to 12.0 mg/mL and reevaluated via size exclusion chromatography periodically to ensure stability of each protein population. The monomer and cross-linked aggregate form were found to be stable for more than 3 months. Although less than 5% of the monomer form did become aggregated after 3 months this does not affect the conclusions drawn since each sample would contain a slightly higher percentage of aggregate.

Purified BSA was obtained from Sigma Aldrich Pharmaceuticals. The protein was then applied to a 5 × 1 cm chromatography column containing charcoal (Fluka, St. Louis Missouri, USA^61^) to remove associated lipids from the protein. This was followed by concentration and application of the purified protein to a Yarra-3000 size exclusion column which enable isolation of monomeric, dimeric and tetrameric BSA. The monomer and different aggregates were concentrated to 15 mg/mL and analyzed over time via DLS to determine and verify stability of the BSA monomer and BSA aggregate populations. The monomer and aggregate forms were found to be stable for more than 3 months. For BSA, less than 3% of the monomer form became aggregated after 3 months. In consequence and as explained for the lysozyme sample, we do not believe this significantly affects our conclusions, since it indicates that each sample ended up containing a slightly higher percentage of aggregates. As a result, there was no need to apply the cross-linking method described for the lysozyme aggregate preparation.

### Fluorescent labeling of protein aggregates

To investigate the amount of impurity incorporated into growing crystals, stable fluorescently labeled protein aggregates were prepared and subsequently added at different percent concentrations to non-labeled monomeric protein suspensions. The *Pf*GST tetramer as well as BSA and lysozyme dimers, acting as related impurities in the crystallization experiments, were labeled with fluorescent dyes Alexa fluor 488, TFP Esther and Alexa fluor 594 and HS Esther respectively. The Alexa fluor 488 TFP Esther and Alexa fluor 594 and HS Esther were obtained from thermal Fisher Scientific (life technology). The Alexa fluor 488 TFP Esther (5 mg) was dissolved in 0.5 mL of dimethyl sulfoxide. The reactive dye solution (80 µL) was slowly added to the stirring *Pf*GST tetramer solution (10 mg/mL in PBS). The reaction was incubated for 1 h at room temperature with continuous stirring. Labeled tetramer was separated from free dye using a DAX G 25 prepack gel filtration column. The amount of dye per mole of protein was determined as described previously^45^, using the molar extinction coefficients, e.g. 71,000 cm^–1^ M^–1^ for Alexa fluor 488 at 494 nm wavelength. The resulting degree of labeling (DOL) for Alexa fluor 488 and *Pf*GST tetramer used in crystallization experiments was determined to 3.8 dye molecules per protein molecule. For lysozyme and BSA aggregate labeling, a solution of the buffered dimeric protein at 2 mg/mL was mixed with Alexa floor 594 and HS Esther at 22 °C and incubated for 1 h to yield a DOL of approx. 2 and 5 for lysozyme and BSA respectively. Unbound dye was removed via spin concentration.

### Crystallization protocols

Crystallization of lysozyme was performed in 50 mM acetate buffer pH 4.8, 100 mM NaCl and protein concentration at 10 mg/mL. Crystallization of BSA was performed at pH 6.5 in 0.1 M MES buffer, a protein concentration of 40 mg/mL and using 24% polyethylene glycol 4000 as precipitant^62^. *Pf*GST crystallization was performed in 0.1 M NaH_2_PO_4_, applying a protein concentration of 30 mg/mL using a solution of 2.8 M (NH_4_)_2_SO_4_, 0.1 M NaH_2_PO_4_, 15% glycerol at pH 6.7 as precipitant.

Crystallization experiments were performed using the counter/liquid- liquid diffusion technique in capillaries (VitroCom, Inc.) of 100 mm length, 3 mm width, and 0.3 mm internal height (Fig. 3). Capillaries were filled with 37 µL of precipitant and 37 µL of protein solution (supplemented with different percentages of fluorescently labeled protein aggregate) using Hamilton syringes. Lysozyme, concentrated to 10 mg/mL, was prepared in 0.1 M sodium acetate (pH 4.6) solution with the precipitant solution consisting of 1 M sodium chloride and 0.1 M sodium acetate adjusted to pH 4.6. Purified and delipidated BSA was prepared in Tris-HCl buffer at pH 7.5. A sample of *Pf*GST dimer and tetramer was prepared in 0.1 M Na_2_HPO_4_ (pH 6.7) solution. The precipitant solution for *Pf*GST consisted of 2.8 M (NH_4_)_2_SO_4_, 0.1 M Na_2_HPO_4_ and 15% glycerol (pH 6.7). Immediately after preparing each capillary the capillaries were immersed in liquid nitrogen to freeze the samples prior to launch. Eight capillaries were contained in a specially constructed cassette (ZIN Technologies) that allows the capillaries to lie flat in the cassette channels thereby enabling optical viewing with the International Space Station’s LMM. It should be noted that it would have been helpful to monitor the time course of crystallization rates in microgravity versus 1 G controls but experimental constraints using the LMM prevented these measurements. Additional details regarding the sample preparation prior to flight are provided in a previous publication^45^. Figure 3 shows one of the flight capillary cassettes containing eight prepared capillaries. For subsequent experiments, crystals of similar shape and size in all three dimensions were selected to maintain comparability for the diffraction data and fluorescence intensities of crystals grown under different gravity conditions. Crystallization capillaries filled with protein and precipitant solution used for unit gravity control experiments were prepared and handled identically as for the microgravity on-orbit experiments to maintain optimal comparability. Notably, all capillaries were instantly flash frozen in liquid nitrogen, as required for transportation, and all ground-control capillaries were stored and thawed on the same day as the respective microgravity sample capillaries.

### On-orbit experiments

Crystallization experiments were performed on the International Space Station (ISS) on two different mission launch dates SPX10, February 19th, 2017 (Lysozyme, *Pf*GST) and SPX15, June 29th, 2018 (BSA, Lysozyme) with samples returned on January 16th, 2019. The SPX10 mission crystal growth duration was 30 days and the SPX15 crystal growth duration was 202 days (for this mission, samples returned via a subsequent SPX16 flight to the ISS). For each mission, the crystallization cassettes were maintained at −80 °C, i.e. contained in a −80 °C freezer) within the SpaceX Dragon module until reaching the ISS. Once on orbit, the freezer was transferred to the ISS and the crystallization cassettes removed from the −80 °C freezers, and allowed to thaw, thereby initiating the liquid-diffusion process. Crystallization growth temperature was maintained between 20–23 °C for both missions. Crystal growth was monitored periodically utilizing the NASA LMM with crystallization images photographed to support measurement of crystal growth rates.

### Confocal fluorescence imaging experiments

To investigate incorporation of impurities into growing crystals for each protein, confocal fluorescence imaging experiments were performed on the ISS using the LMM microscope, ×2.5 and ×10 objectives. Fluorescence imaging experiments on the ground were performed using a Zeiss LSM710 confocal laser scanning microscope (Carl Zeiss microscopy). The Laser Confocal Microscope uses discrete laser excitation to acquire haze free high-resolution images at specific, spectral, wavelengths using PMT detectors at a resolution of 1024 × 1024 pixels. Through motorized focus control, the Zeiss ZEN imaging software acquires multiple Z-axis plane images and reconstructs and renders 3D images from the planes. These reconstructed 3D z-stacks can be used to calculate the mean intensity of each crystal. The Nis-Elements Analysis Software package has an automated measurement feature that can be used to select specific 3D regions in an image. The mean intensity distribution on a defined crystal volume can be measured and recorded and then exported to Excel. This allows the investigator to compare intensity of an equal volume for each crystal thereby enabling accurate comparison of microgravity versus ground-control crystals. Further, crystals of similar size were selected for comparison. Fluorescence investigation of *Pf*GST crystals used a FITC (excitation 475–495 nm, emission 515–545 nm) filter and for lysozyme and BSA crystals, a Texas Red (excitation 540–580 nm, emission 590–630 nm) filter was used.

### X-ray diffraction data collection and processing

For cryo-protection prior to shock-freezing needle-shaped *Pf*GST crystals were carefully harvested from the capillaries and embedded in cryoprotectant solution (0.1 M NaH_2_PO_4_, 1.9 M (NH_4_)_2_SO_4_, 10 mM *S*-(*p*-bromobenzyl)-GSH, 14% glycerol, pH 6.7) using nylon loops of identical size (Mounted Cryo-Loops, Hampton Research, US). Diffraction data of individual crystals with homologous dimensions of approx. 200 µm in length were collected using a constant instrumentation setup containing a PILATUS 6 M detector at the EMBL beamline P13 and beamline P11 (PETRA III, DESY, Hamburg, Germany) at 100 K, collecting 3600 images per crystal with 0.1° rotation increment. Diffraction data were subsequently indexed, integrated and scaled using XDS and evaluated. Crystal parameters and X-ray diffraction statistics are summarized in Tables 1 and 2. Three datasets were recorded and compared for each condition.

### Reporting summary

Further information on research design is available in the Nature Research Reporting Summary linked to this article.

## Supplementary information

Reporting Summary

## Data Availability

All data collected for the fluorescence experiments, as well as for the X-ray diffraction experiments can be obtained from the authors on request. Correspondence should be addressed to L.J.D. and C.B.
